# High‐throughput isolation of circulating tumor DNA: a comparison of automated platforms

**DOI:** 10.1002/1878-0261.12415

**Published:** 2018-12-22

**Authors:** Lisanne F. van Dessel, Silvia R. Vitale, Jean C. A. Helmijr, Saskia M. Wilting, Michelle van der Vlugt‐Daane, Esther Oomen‐de Hoop, Stefan Sleijfer, John W. M. Martens, Maurice P. H. M. Jansen, Martijn P. Lolkema

**Affiliations:** ^1^ Department of Medical Oncology Erasmus MC Cancer Institute University Medical Center Rotterdam The Netherlands; ^2^ Workgroup Cancer Genomics Netherlands Erasmus MC Cancer Institute University Medical Center Rotterdam The Netherlands; ^3^ Department of Clinical and Experimental Medicine Center for Experimental Oncology and Hematology University of Catania Italy

**Keywords:** automation, cell‐free DNA, circulating tumor DNA, isolation

## Abstract

The emerging interest in circulating tumor DNA (ctDNA) analyses for clinical trials has necessitated the development of a high‐throughput method for fast, reproducible, and efficient isolation of ctDNA. Currently, the majority of ctDNA studies use the manual QIAamp (QA) platform to isolate DNA from blood. The purpose of this study was to compare two competing automated DNA isolation platforms [Maxwell (MX) and QIAsymphony (QS)] to the current ‘gold standard’ QA to facilitate high‐throughput processing of samples in prospective trials. We obtained blood samples from healthy blood donors and metastatic cancer patients for plasma isolation. Total cell‐free DNA (cfDNA) quantity was assessed by TERT quantitative PCR. Recovery efficiency was investigated by quantitative PCR analysis of spiked‐in synthetic plant DNA. In addition, a β‐actin fragmentation assay was performed to determine the amount of contamination by genomic DNA from lysed leukocytes. ctDNA quality was assessed by digital PCR for somatic variant detection. cfDNA quantity and recovery efficiency were lowest using the MX platform, whereas QA and QS showed a comparable performance. All platforms preferentially isolated small (136 bp) DNA fragments over large (420 and 2000 bp) DNA fragments. Detection of the number variant and wild‐type molecules was most comparable between QA and QS. However, there was no significant difference in variant allele frequency comparing QS and MX to QA. In summary, we show that the QS platform has comparable performance to QA, the ‘gold standard’, and outperformed the MX platform depending on the readout used. We conclude that the QS can replace the more laborious QA platform, especially when high‐throughput cfDNA isolation is needed.

**Abbreviations**
cfDNA: cell‐free DNA
cRNA: carrier RNA
ctDNA: circulating tumor DNA
dPCR: digital PCR
HBD: healthy blood donor
MX: Maxwell
QA: QIAamp
qPCR: quantitative PCR
QS: QIAsymphony
SNP: single nucleotide polymorphism
VAF: variant allele frequency

## 1 Introduction

With the discovery of cell‐free DNA (cfDNA), first described in 1948 by Mandel and Metais (1948), and subsequently circulating tumor DNA (ctDNA; Stroun *et al*., 1989), a novel biomarker in cancer research became available. Since then, many studies have shown its great potential for detecting minimal residual disease and evaluating treatment response (Bidard *et al*., 2014; Dawson *et al*., 2013; Diaz and Bardelli, 2014; Diehl *et al*., 2008; Forshew *et al*., 2012; Herbreteau *et al*., 2018; Murtaza *et al*., 2013; Pugh, 2018; Shinozaki *et al*., 2007). However, to enable high‐throughput ctDNA analyses a fast, accurate, and efficient cfDNA isolation method is highly needed.Currently, the majority of ctDNA studies use Qiagen's QIAamp (QA) platform for cfDNA isolation (Oxnard *et al*., 2014; Sefrioui *et al*., 2015; Zill *et al*., 2015). However, this manual platform is laborious and can only process up to 24 samples at a time rendering this method less suitable for large‐scale studies. Automation of cfDNA isolation represents a potential solution provided that it is able to (a) reduce hands‐on time; (b) simultaneously process large numbers of samples; (c) accurately and reproducibly isolate cfDNA with a reasonable recovery; and (d) preserve the quality of ctDNA for downstream analyses.Cell‐free DNA is naturally fragmented (140–175 bp) and only present at low concentrations in the blood circulation (usually around 10 ng per mL plasma; Fleischhacker and Schmidt, 2007). In addition, the fraction of ctDNA relative to cfDNA can vary from extremely low (< 0.01%) to very high (60%), as it is dependent on tumor type and stage (Bettegowda *et al*., 2014; Diehl *et al*., 2008). Together these features make it imperative to carefully determine the efficacy of DNA isolation instead of merely investigating isolation yields. Furthermore, isolation of cfDNA and ctDNA therein is highly susceptible to genomic DNA contamination from lysed leukocytes (Elshimali *et al*., 2013; Jahr *et al*., 2001), resulting in a potential underestimation of the ctDNA fraction and decreasing the detection sensitivity. As potential differences in cfDNA recovery efficiency between isolation methods might affect downstream analysis results of ctDNA by decreasing its detection sensitivity, standardized comparison of the different methods for cfDNA isolation is important and highly needed.The purpose of this study was to compare two automated cfDNA isolation platforms, Maxwell (MX) and QIAsymphony (QS), to the current ‘gold standard’ QA isolation kit to determine whether these automated platforms can facilitate high‐throughput processing of samples in prospective trials. Our analyses focused on both qualitative and quantitative parameters, including cfDNA yield, recovery efficiency, cfDNA fragmentation patterns, and ctDNA fraction retrieved, using optimally processed plasma samples of healthy blood donors (HBDs) and patients with metastatic cancer.

## 2 Materials and methods

### 2.1 Subjects

Blood samples were obtained from a total of 10 HBDs and 10 metastatic cancer patients. HBDs were either laboratory volunteers or blood donors of the Sanquin Blood Bank South‐West Region, The Netherlands. Patients were enrolled in this study between September 2016 and September 2017 within the Erasmus MC Cancer Institute in Rotterdam, the Netherlands. Eligibility criteria for patients have been described previously (van Dessel *et al*., 2017). All patients provided written informed consent, and the institutional review board approved the protocols (Erasmus MC ID MEC 15‐616). The study methodologies conformed to the standards set by the Declaration of Helsinki. Patient and tumor characteristics are summarized in Table 1.

**Table 1:** Patient and tumor characteristics

Patient ID (#)	Primary tumor	Known somatic variant (nucleotide change)	Variant allele frequency in tissue (%)
BP‐001	NSCLC	KRAS p.G12C (c.34G>T)	32
BP‐003	Melanoma	NRAS p.Q61R (c.182A>G)	88
BP‐004	Melanoma	BRAF p.V600E (c.1799_1800delinsAA)	50
BP‐007	Melanoma	BRAF p.V600K (c.1798_1799delGTinsAA)	38
BP‐008	CRC	KRAS p.G12D (c.35G>A)	45
BP‐009	CRC	PIK3CA p.E545K (c.1633G>A)	45
BP‐015	CRC	KRAS p.G13D (c.38G>A)	40
BP‐016	CRC	KRAS p.G12V (c.35G>T)	Unknown
BP‐023	CRC	KRAS p.G13D (c.38G>A)	Unknown
BP‐028	Melanoma	BRAF p.V600K (c.1798_1799delinsAA)	55

CRC, colorectal cancer; NSCLC, non‐small‐cell lung cancer.

### 2.2 Blood collection

Healthy blood donors donated 20 mL of blood, collected either in 2 × 10 mL CellSave preservative tubes (Janssen Diagnostics, Raritan, NJ, USA) or in 1 × 10 mL EDTA tube (Becton, Dickinson and Company, Franklin Lakes, NJ, USA) and 1 × 10 mL CellSave preservative tube. Patients donated 3 × 10 mL of blood collected in CellSave preservative tubes. Blood samples were stored at room temperature until further processing. After blood draw, samples in EDTA tubes were processed within 24 h, whereas samples in CellSave tubes were processed within 96 h for plasma isolation as previously described (van Dessel *et al*., 2017).

### 2.3 cfDNA isolation

Cell‐free DNA was isolated from 2 mL of plasma and eluted in 60 μL of the provided elution buffer. Three isolation platforms were evaluated (Table 2):

- QIAamp® (QA) Circulating Nucleic Acid Kit (Qiagen, Hilden, North Rhine‐Westphalia, Germany);
- QIAsymphony® (QS) SP Circulating DNA Kit (Qiagen);
- Maxwell® (MX) RSC LV ccfDNA Plasma Custom Kit (Promega, Madison, WI, USA).

**Table 2:** Specifications of cell‐free DNA isolation platforms

Platform	Manufacturer	Protocol	cfDNA isolation kit	Plasma input (mL)	Number of samples per run	Handling time per run (min)	Technique	Cost (€) per sample
QIAamp (QA)	Qiagen	Manual	QIAamp® Circulating Nucleic Acid Kit	1.0–5.0	24	180–240	Vacuum‐column‐based	20
QIAsymphony (QS)	Qiagen	Automatic	QIAsymphony® Circulating DNA Kit	2.0–8.0^a^	96	30	Magnetic‐bead‐based	24
Maxwell (MX)	Promega	Automatic	Maxwell® RSC LV ccfDNA Plasma Custom Kit	2.0–4.0^a^	16^b^	30	Magnetic‐bead‐based	20

a Upon request, the manufacturer is able to adjust system settings and protocols for lower/higher plasma input volumes.

b The Maxwell RSC 48 Instrument can process up to 48 samples per run.

All cfDNA isolations were performed according to the manufacturer's protocol, with some minor modifications. In more detail, cfDNA was isolated with QA as previously described (van Dessel *et al*., 2017). The QS isolation was adapted by adding 1 μg of carrier RNA (cRNA, Qiagen) to the plasma sample preceding isolation. Using the MX platform, a third plasma centrifugation step at 2000 ***g*** for 10 min at room temperature was performed after thawing to eliminate residual leukocytes, as recommended by the manufacturer. The custom Maxwell® RSC ccfDNA Plasma Kit for large plasma volume protocol was used. In brief, 2 mL of plasma was added to an equal amount of binding buffer and 140 μL of magnetic beads. This mixture was incubated under rotation for 45 min at room temperature and subsequently centrifuged at 2000 ***g*** for 1 min at room temperature. The pelleted mix of beads and cfDNA was then transferred to the cartridge and run on the MX instrument (Promega) according to the manufacturer's protocol.

### 2.4 Testing of cRNA addition to the automated platforms

Plasma samples from several HBDs were pooled and divided into aliquots of 2 mL each. To each aliquot, we added different amounts of cRNA, ranging from 0.25 up to 4 μg. As a control, plasma samples without cRNA were included. To allow determination of the recovery efficiency, synthetic plant DNA was added to plasma samples (see below).

### 2.5 cfDNA quantification

All cfDNA samples were quantified by both Qubit™ fluorometric quantitation (Invitrogen, Life Technologies, Carlsbad, CA, USA) and human TaqMan® copy number reference assay TERT (Applied Biosystems, Life Technologies, Foster City, CA, USA) by quantitative PCR (qPCR). The Qubit™ measurement was performed on 2 μL of each cfDNA sample using the Quant‐iT dsDNA high‐sensitivity assay (Invitrogen), according to the manufacturer's protocol. TERT qPCRs contained 5 μL cfDNA, 3.13 μL SensiFAST™ SYBR® Lo‐Rox mix (Bioline, London, UK), and 0.62 μL TERT assay in a total reaction volume of 12.5 μL. The qPCR was performed on an Mx3000P Real‐Time PCR System (Agilent, Santa Clara, CA, USA) with a pre‐incubation at 95 °C for 10 min, followed by 45 cycles of 95 °C for 10 s and 60 °C for 22 s. cfDNA was quantified using a standard curve of human genomic DNA.

### 2.6 Synthetic plant DNA and plant DNA qPCR assay

The synthetic plant DNA assay developed by Kang *et al*. (2016) was used as an exogenous control to calculate the recovery efficiency of each cfDNA isolation method. In short, 250 ng of a 150‐bp gBlocks® gene fragment (Integrated DNA Technologies Incorporation (IDT), Coralville, IA, USA) was resuspended in LoTE buffer to a final concentration of 1.64x10^0^ ng·μL^−1^. The stock sample was serially diluted to a final concentration of 1.64 × 10^−6^ ng·μL^−1^ of which 5 μL was spiked into plasma preceding cfDNA isolation. Plant DNA qPCRs were essentially performed as described above, using 900 nm of both forward and reverse primer and 250 nm of a FAM‐labeled probe (Table S1). Recovery efficiency was determined using a standard curve including the amount of spiked‐in plant DNA. Samples with a recovery efficiency < 5% or > 100% were excluded from further analysis as this strongly suggested an operator failure. This was further supported by the fact that recovery efficiency was not strongly correlated (ρ = 0.45) with cfDNA concentration (Fig. S1).

### 2.7 Digital PCR TaqMan® SNP genotyping and β‐actin fragmentation assay

The presence of somatic tumor‐specific variants and wild‐type DNA molecules was determined using standard and custom‐made TaqMan® single nucleotide polymorphism (SNP) genotyping assays (Thermo Fisher Scientific, Waltham, MA, USA), according to the manufacturer's instructions (Tables S2 and S3). The TaqMan® β‐actin assay was used to investigate the fragment size distribution as an indication of leukocyte DNA contamination of the cfDNA, as previously reported (van Dessel *et al*., 2017). In short, a standard amount of 2 ng of cfDNA was used to detect one small (136 bp) and two long (420 and 2000 bp) β‐actin fragments within a single reaction. The used primers and probes are indicated in Table S1. The digital PCR (dPCR) was performed as previously described (van Dessel *et al*., 2017). In short, a maximum volume input of 7.8 μL of the final cfDNA eluate was added to the dPCR; the dPCR run was performed on the chip‐based QuantStudio 3D Digital PCR System (Thermo Fisher Scientific) according to the manufacturer's protocol. SNP genotyping assays were run at 56 °C; the β‐actin assay was run at 60 °C. A negative control (H_2_O) and a positive control (cell genomic DNA with known variant) were added to every experiment.

### 2.8 Sample size

To test whether QS and MX were comparable to QA, we assumed a Cohen's effect size of 0.8, to be able to detect relevant differences. With a two‐sided type I error probability (α) of 0.025 and a type II error probability (β) of 0.2, a power calculation determined that 18 subjects were needed for paired comparisons. Based on the foregoing, 20 subjects were included (10 HBDs and 10 patients).

### 2.9 Calculations and statistical analysis

All assay results were corrected for variations in plasma input and eluate volume, as previously described (van Dessel *et al*., 2017), and expressed as either ng·mL^−1^ plasma or as mutant/wild‐type/β‐actin copy number per mL of plasma. The variant allele frequency (VAF) was calculated as follows: VAF = total variant copy number/(total variant copy number + total wild‐type copy number).The statistical analyses and figure plotting were performed in R version 3.2.3. The Friedman test was used to test the difference between matched QA, MX, and QS samples. Significant differences were post hoc analyzed using the Wilcoxon signed‐rank test. To correct for multiple testing, we adjusted the *P* value for significance by subsequently applying the Bonferroni correction. The Wilcoxon signed‐rank test was used to test the difference between matched EDTA and CellSave samples. Correlations were determined by Spearman's rank correlation coefficient.

## 3 Results

### 3.1 Optimization of cfDNA isolation using automated isolation platforms

**Figure 1:**
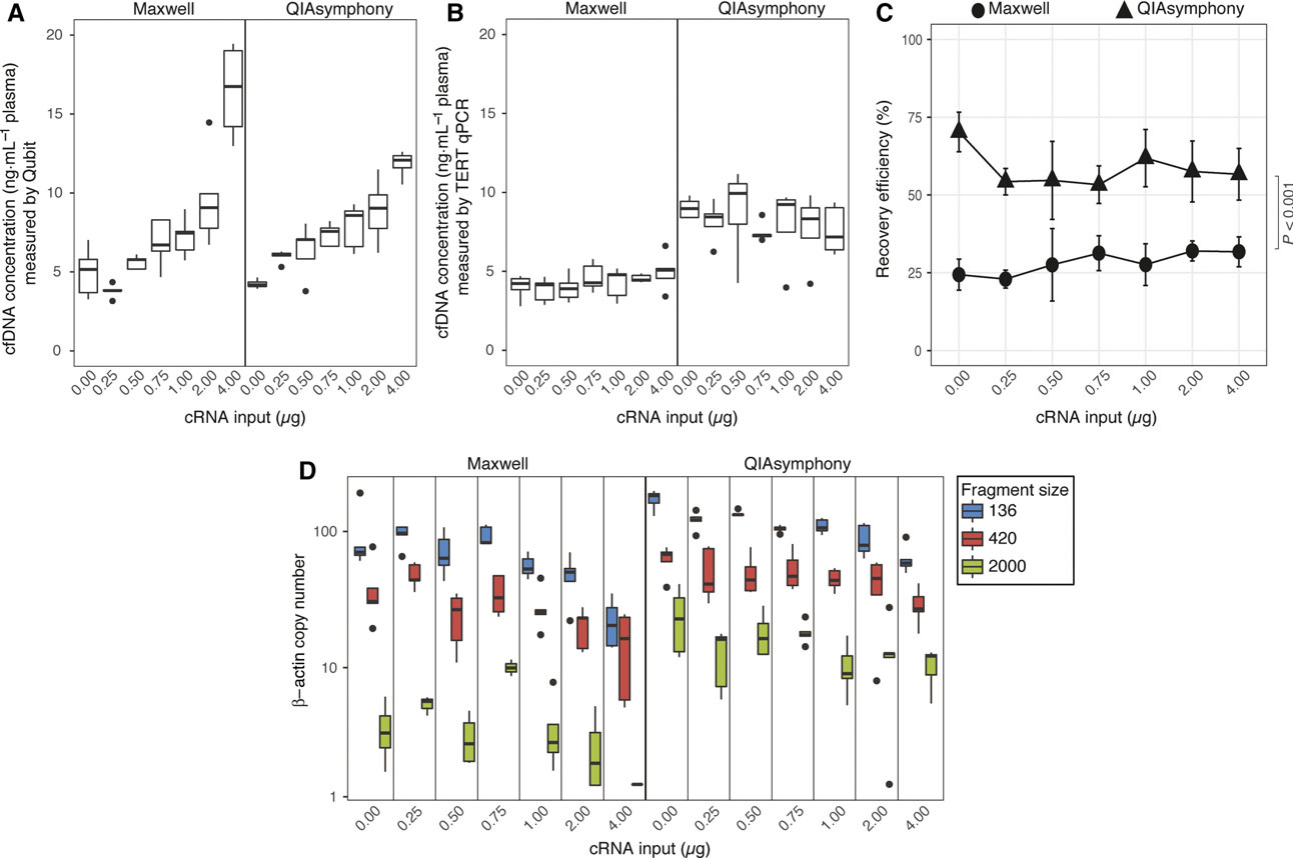
Effect of increasing cRNA input (0–4 μg) on cfDNA quantity and quality using the Maxwell and QIAsymphony platforms. The effect on cfDNA concentration (ng·mL ^−1^ plasma) was measured by Qubit (A) and TERT qPCR (B). The recovery efficiency of each platform was analyzed by qPCR using spiked‐in synthetic plant DNA (C). Differences in cfDNA fragment size, expressed as number of β‐actin fragments for each fragment size (136, 420 and 2000 bp), were analyzed by dPCR (D). Boxes (interquartile ranges; IQR) and whiskers (1.5× IQR) are shown together with the median (black horizontal line). Outliers are indicated as single black points. Symbols ● and ▲ are mean values shown with whiskers (standard deviation). The Friedman test was used to test the group difference between Maxwell and QIAsymphony samples. Significant differences were post hoc analyzed using the Wilcoxon signed‐rank test. *N* = 5.

In a small pilot study, we had previously observed a beneficial effect of cRNA addition to HBD plasma during isolation with the QS protocol on the cfDNA yield as determined by Qubit (Fig. S2). Therefore, cRNA addition was implemented in our standard QS protocol. However, it has been reported that cRNA might interfere with Qubit‐based DNA quantification and might not be a reliable readout (Invitrogen, 2016). Therefore, we tested whether cfDNA isolation on the automated platforms (QS/MX) was beneficially or adversely affected by the addition of cRNA using multiple readouts. We added varying amounts of cRNA to the plasma samples and measured the resulting cfDNA concentration by Qubit and TERT qPCR for both automated platforms**.** Using Qubit as readout, the addition of cRNA increased the total amount of cfDNA extracted on both platforms (MX *P* < 0.001; QS *P* < 0.001; Fig. 1A). However, using TERT qPCR as readout, this increase could not be reproduced (Fig. 1B). Next, we assessed the impact of cRNA on the recovery of spiked‐in synthetic plant DNA. Addition of cRNA affected the recovery efficiency of plant DNA (MX *P* = 0.02; QS *P* = 0.04; Fig. 1C). Independent of cRNA input, recovery of plant DNA was ~ 30% higher with QS (58.37 ± 9.52) than with MX (28.22 ± 6.67; *P* < 0.001). To assess whether the addition of cRNA biased the isolation of particular cfDNA fragment sizes, we performed the β‐actin fragmentation assay (Fig. 1D). For both methods, increasing amounts of cRNA reduced the number of small fragments (136 bp; MX *P* = 0.001; QS *P* < 0.001), while no effect on larger fragments was observed. For all post hoc analyses, paired testing of samples with and without addition of cRNA (0 μg) did not reveal any significant differences.

### 3.2 Compatibility of CellSave preservative tubes with different isolation platforms

**Figure 2:**
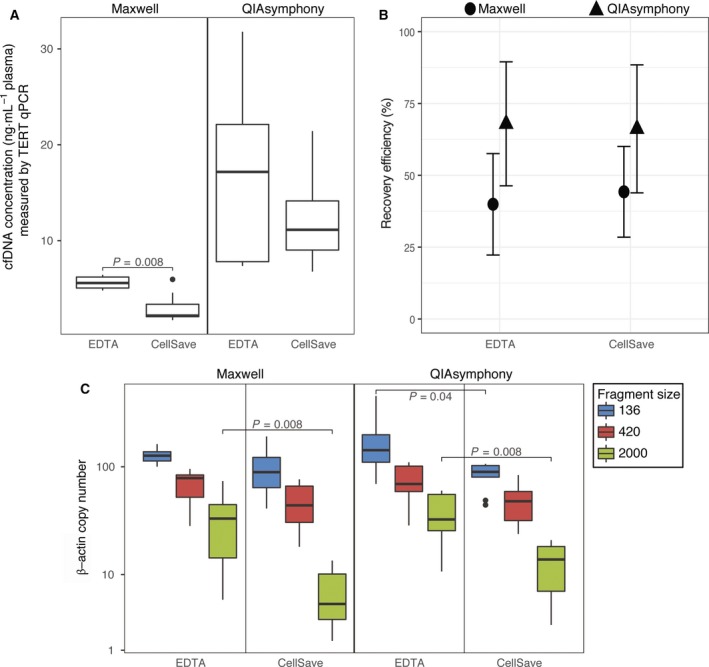
Compatibility of EDTA and CellSave blood collection tubes with the Maxwell and QIAsymphony platforms. The effects on cfDNA concentration (ng·mL ^−1^ plasma) measured by TERT qPCR (A), recovery efficiency measured by plant DNA qPCR (B), and β‐actin fragmentation assay analyzed with dPCR are shown (C). Boxes (interquartile ranges; IQR) and whiskers (1.5× IQR) are shown together with the median (black horizontal line). Outliers are indicated as single black points. Symbols ● and ▲ are mean values shown with whiskers (standard deviation). The Wilcoxon signed‐rank test was used to test the difference between blood collection tubes for each platform. *N* = 9.

Previously, we have demonstrated the good performance of CellSave preservative tubes for ctDNA analysis (van Dessel *et al*., 2017). However, the manufacturers of both automated platforms recommend to use plasma isolated from blood collected in EDTA tubes. To allow for a fair comparison with our CellSave QA results, we therefore first determined whether the automated platforms (QS/MX) were compatible with CellSave tubes by assessing the cfDNA quantity and quality.Figure 2A shows cfDNA concentrations as measured by TERT qPCR analysis. For the MX platform, the median cfDNA concentration was 5.59 ng·mL^−1^ plasma from EDTA tubes and was 2.19 ng·mL^−1^ plasma from CellSave tubes (IQR: 5.06–6.21 and 2.07–3.37 ng·mL^−1^ plasma, respectively; *P* = 0.008). For the QS platform, the median cfDNA concentration was 17.17 ng·mL^−1^ plasma from EDTA tubes and 11.13 ng·mL^−1^ plasma from CellSave tubes (IQR: 7.81–22.12 and 9.02–14.14 ng·mL^−1^ plasma, respectively). Although this was comparable, EDTA samples displayed a larger range in yielded cfDNA concentration. The potential effect of CellSave tubes on the recovery of synthetic plant DNA was determined as well. Comparable recovery efficiencies were observed in plasma collected in EDTA and CellSave tubes for both platforms (39.92% vs. 44.27% in MX and 67.92% vs. 66.19% in QS; Fig. 2B). Finally, we used the β‐actin fragmentation assay to evaluate cfDNA fragmentation patterns as a readout for general sample quality (Fig. 2C). EDTA tubes yielded a higher number of large cfDNA fragments (2000 bp) irrespective of the platform used (median number of β‐actin fragments and IQR MX: 33.08 (14.28–44.59); QS: 32.46 (25.53–55.44)) than CellSave tubes (median number of β‐actin fragments and IQR MX: 5.15 (2.42–9.17); QS: 13.80 (7.01–18.18); *P* = 0.008). The number of small DNA fragments (136 bp) did not differ between EDTA and CellSave tubes for MX, but was slightly higher for EDTA tubes on the QS platform (median number of β‐actin fragments and IQR EDTA: 142.71 (110.28–198.18); CellSave: 89.71 (80.22–102.64); *P* = 0.04). Based on these results, we deemed CellSave tubes are compatible with both automated platforms and used them for all further experiments.

### 3.3 Comparison of the performance of automated platforms on downstream cfDNA and ctDNA analyses

**Figure 3:**
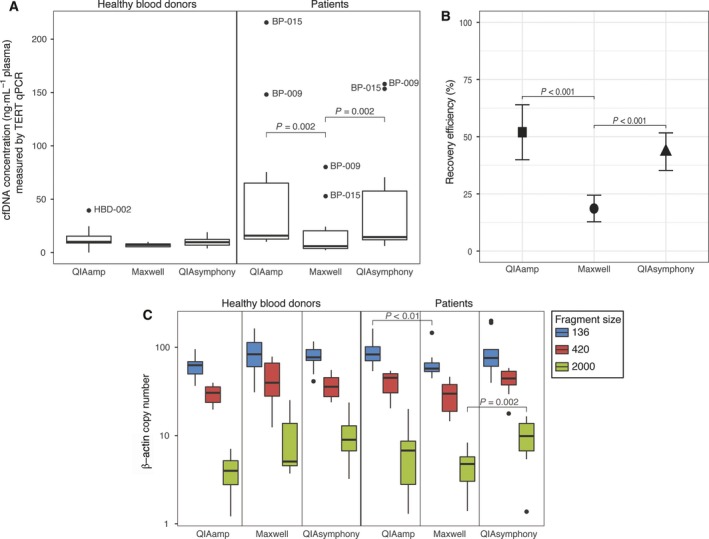
Effect of the different isolation platforms (QIAamp, Maxwell, and QIAsymphony) on downstream cfDNA analysis. cfDNA was isolated from 2 mL matched plasma samples of HBDs (*N* = 10) and patients with metastatic cancer (*N* = 10) and analyzed by TERT qPCR assay for cfDNA concentration (ng·mL ^−1^ plasma) (A), plant DNA qPCR assay to determine recovery efficiency (B), and dPCR β‐actin fragmentation assay to evaluate cfDNA fragment sizes (C). Boxes (interquartile ranges; IQR) and whiskers (1.5× IQR) are shown together with the median (black horizontal line). Outliers are indicated as single black points. Symbols ■, ●, and ▲ are mean values shown with whiskers (standard deviation). The Friedman test was used to test the group difference between matched samples processed by the three platforms. Significant differences were post hoc analyzed using the Wilcoxon signed‐rank test.

Next, we compared the quantity and quality of the obtained cfDNA using the current ‘gold standard’ manual QA platform to the automated QS and MX platforms using samples from 10 HBDs and 10 metastatic cancer patients. In HBDs, cfDNA concentrations measured by TERT qPCR analysis were comparable for all three isolation platforms (Fig. 3A). In patients, the MX retrieved significantly less cfDNA compared to both QA (*P* = 0.002) and QS (*P* = 0.002; median cfDNA concentration and IQR QA: 15.84 (12.64–65.11); MX: 6.00 (3.80–20.43); QS: 14.50 (11.99–57.65) ng·mL^−1^ plasma; Fig. 3A). To determine the recovery efficiency of the three different platforms, 5 μL of synthetic plant DNA was added to each plasma sample preceding cfDNA isolation. The average recovery efficiency using QA (51.95 ± 12.02%) was similar to QS (43.45 ± 8.21%). However, MX performed worse (18.61 ± 5.81%; *P* < 0.001; Fig. 3B). In HBDs, we did not observe cfDNA fragment size differences between either of the evaluated platforms (Fig. 3C). In patients, MX isolated fewer small β‐actin fragments (136 bp) than QA (median number of β‐actin fragments and IQR for MX: 57.45 (53.17–66.72); and for QA: 83.18 (70.36–101.63); *P* < 0.01) and fewer large fragments (2000 bp) than QS (median number of β‐actin fragments and IQR for MX: 2.08 (0.00–5.21); and for QS: 10.06 (6.70–13.72); *P* = 0.002).

**Figure 4:**
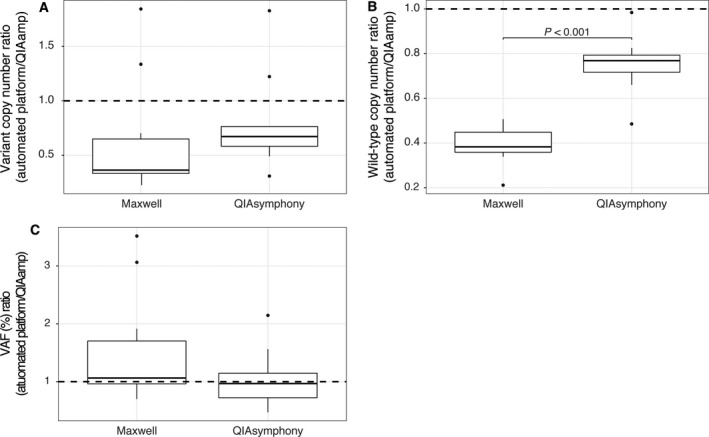
Somatic variant detection in patients with metastatic cancer on samples isolated with the three different isolation platforms (QIAamp, Maxwell, and QIAsymphony). Somatic variant status had been assessed in patients’ primary and/or metastatic lesion as part of the standard of care. In all patients (*N* = 10), the known somatic variant was detected in plasma isolated from the three platforms. The ratios of the mutant copy number (A), wild‐type copy number (B), and variant allele frequency (VAF) measured in the Maxwell and QIAsymphony vs. QIAamp are shown (C). The dashed line (ratio of 1) resembles the situation when platforms have similar results. The Wilcoxon signed‐rank test was used to test the difference between the platforms.

Finally, we compared somatic variant detection in ctDNA isolated by the different platforms. For this purpose, we used previously generated diagnostic sequencing results on the somatic variant status in the primary and/or metastatic lesions of the corresponding patients (Table 1). We detected the expected somatic variants in all patients for all isolation methods. QS results were most comparable to QA (Fig. 4). In MX, fewer mutant molecules, though not significant, and significantly fewer wild‐type molecules were isolated (Fig. 4A,B). However, this did not result in a significantly different VAF (Fig. 4C).

## 4 Discussion

Up to now, several studies have investigated the effect of manual and automated cfDNA isolation platforms on ctDNA quantity and quality (Devonshire *et al*., 2014; Perez‐Barrios *et al*., 2016; Sorber *et al*., 2017). However, differences in pre‐analytical conditions, including plasma processing time, type of blood collection tube used, and storage conditions, hamper direct comparisons and straightforward conclusions. Here, we presented a study in which we have systematically optimized and compared automated isolation of cfDNA using QS and MX with the ‘gold standard’ QA.The addition of carrier molecules like cRNA to plasma preceding cfDNA isolation increases the amount of cfDNA recovered during isolation by precipitating and binding of small molecules (Kishore *et al*., 2006; Shaw *et al*., 2009). The manual QA platform requires addition of cRNA for the standard protocol, whereas the manufacturer's protocol of both the QS and MX does not require this. In a small pilot study, we observed that the addition of cRNA to the QS protocol improved cfDNA yield, so cRNA was implemented into our standard QS protocol. However, Invitrogen has reported that cRNA might interfere with Qubit‐based DNA quantification. Indeed, our findings suggest that the increase in cfDNA concentration as measured by Qubit for QS and MX is, at least in part, affected by the presence of cRNA. Data obtained from the TERT and plant DNA qPCR did not reveal any added value of cRNA to either of the automated platforms. Moreover, our fragmentation assay suggests that increasing amounts of cRNA reduce the amount of small fragments. Together, our results demonstrate that addition of cRNA to plasma does not improve cfDNA yields using these automated bead‐based platforms.In our previous study using the manual QA platform, we demonstrated the superiority of CellSave tubes over EDTA tubes for collecting plasma for cfDNA/ctDNA analysis as it ensures optimal ctDNA quality when processed within 96 h after blood draw compared to only 24 h for EDTA tubes, enabling its use in multicenter clinical studies (van Dessel *et al*., 2017). Therefore, we investigated the compatibility of CellSave tubes with QS and MX. On both platforms, we observed an increase in the isolation of large cfDNA fragments (2000 bp) in EDTA samples. This relates to the release of intact DNA from lysed leukocytes and a subsequent increase in cfDNA concentration, which we also observed here. As the recovery efficiency was not affected in CellSave tubes and the plasma samples were not contaminated with additional DNA from leukocytes, we recommend the use of CellSave tubes in combination with the QS or MX platform.Currently, QA is widely used for cfDNA/ctDNA isolations, but its manual laborious and time‐consuming protocol renders this method unsuitable for high‐throughput isolations. The competing automated platforms QS and MX both use magnetic‐bead‐based protocols and have comparable hands‐on times. However, costs and number of samples that can be processed per run differ (Table 2). In HBDs, cfDNA quantity and quality were similar on all platforms. However, in patients we saw for all assays that QA and QS yielded more cfDNA than MX. As this might suggest that higher amounts of cfDNA are less efficiently isolated by the MX platform, we spiked high amounts of fragmented DNA in HBD plasma and isolated this with MX (Fig. S3). However, these high DNA amounts were isolated efficiently by MX. Another potential explanation for the difference in performance might be the absence of proteinase K incubation step in the MX protocol. Proteinase K is used in both the QA and QS protocols and can improve cfDNA yield by inhibiting nucleases and the release of protein‐bound cfDNA. Moreover, recovery efficiency of plant DNA was lowest in MX. Altogether, this explains the lower yield of mutant and wild‐type molecules isolated by MX, which may be a concern in samples with low frequent somatic variants. However, importantly, this lower yield did not translate into a significant difference in detected VAF (Figs 4C and S4). These data underline the importance of taking the used isolation method and readout (mutant molecules·mL^−1^ plasma or VAF) into consideration when comparing results between studies as well as for the diagnostic use of ctDNA. QS and QA performed comparable in detection of absolute numbers of mutant and wild‐type molecules. Of note, other publications have observed similar performances of QA and MX in a head‐to‐head comparison (Perez‐Barrios *et al*., 2016; Sorber *et al*., 2017). This could be related to differences in pre‐analytical conditions (e.g., type of blood collection tube, plasma volume used as input), as multiple publications have demonstrated its relation to cfDNA quantity and quality (Haselmann *et al*., 2018; van Dessel *et al*., 2017; Volckmar *et al*., 2018). In addition, we have optimized our QA protocol by re‐eluting three times and thereby improving our cfDNA quantity. For automated magnetic‐bead‐based systems, this is not possible.

## 5 Conclusion

The results of this study show that the QS automated platform has comparable performance to the ‘gold standard’ QA and outperformed the MX platform depending on the readout used. The QS platform is congruent with all our predefined goals as it (a) reduces hands‐on time from 180–240 to 30 min per run; (b) is able to process larger numbers of samples (96 instead of 24 at a time); (c) isolates comparable cfDNA yield with similar efficiency; and (d) has comparable ctDNA quantity and quality to QA. Therefore, the QS can replace the more laborious QA platform, especially when high‐throughput cfDNA isolation is needed.

## Conflict of interest

The authors declare no conflict of interest.

## Author contributions

LFD and SRV acquired data, analyzed and interpreted data, and drafted and revised the manuscript; JCAH and MVD acquired data and revised the manuscript; SMW analyzed and interpreted data and revised the manuscript; EOH carried out statistical analyses and revised the manuscript; SS, JWMM, and MPL conceived the project and revised the manuscript; MPHMJ analyzed and interpreted data, conceived the project, and revised the manuscript; and all authors read, critically revised, and approved the final manuscript.

## Supporting information


**Fig. S1.** Overview of the recovery efficiency of synthetic plant DNA in all samples isolated with the different platforms (QA, MX, and QS). (A) Dot plot of the recovery efficiency for each isolation platform, as analyzed by plant qPCR using spiked‐in synthetic plant DNA. Samples with a recovery efficiency < 5% or > 100% (black horizontal lines) were excluded from the analyses. (B) Correlation between recovery efficiency and cfDNA concentration (ng·mL^−1^ plasma) measured by TERT qPCR assay. Correlations were tested by Spearman's rank correlation coefficient. *P < 0.001.

**Fig. S2.** Effect of cRNA addition on cfDNA quantity using the QS platform. cfDNA concentration (ng·mL^−1^ plasma) was determined by Qubit after adding increasing amounts of cRNA (0–4 μg) before start of the plasma isolation. Boxes (interquartile ranges; IQR) and whiskers (1.5× IQR) are shown together with the median (black horizontal line).

**Fig. S3.** Performance of the MX platform using increasing DNA input (0, 15, and 60 ng·mL^−1^ fragmented cell line DNA has been spiked in HBD plasma). The effects on (A) cfDNA concentration (ng·mL^−1^ plasma) measured by TERT qPCR, (B) recovery efficiency measured by plant DNA qPCR, (C) total number of mutant molecules, and (D) VAF are shown. Boxes (interquartile ranges; IQR) and whiskers (1.5× IQR) are shown together with the median (black horizontal line). Outliers are indicated as single black points. Symbol ● is mean value shown with whiskers (standard deviation). *N* = 5.

**Fig. S4.** Representative data images of SNP genotyping dPCR assay isolated with the different platforms (QA, MX, and QS). A subject with an intermediate (A), high (B) and low (C) VAF are shown. On the *Y*‐axis, positive FAM signal represents mutant molecules (blue dots); on the *X*‐axis, positive VIC signal represents wild‐type molecules (red dots). Green dots reflect the presence of a mutant and a wild‐type molecule in a single well.

**Table S1.** Custom primer and probe sequences used for qPCR.
**Table S2.** Standard SNP genotyping assays.
**Table S3.** Custom SNP genotyping assays.
