# Supplementary figures and images for: High‐throughput isolation of circulating tumor DNA: a comparison of automated platforms
Source: Mol Oncol. 2018 Dec 22;13(2):392–402. doi: 10.1002/1878-0261.12415 (PMC6360376; doi:10.1002/1878-0261.12415)

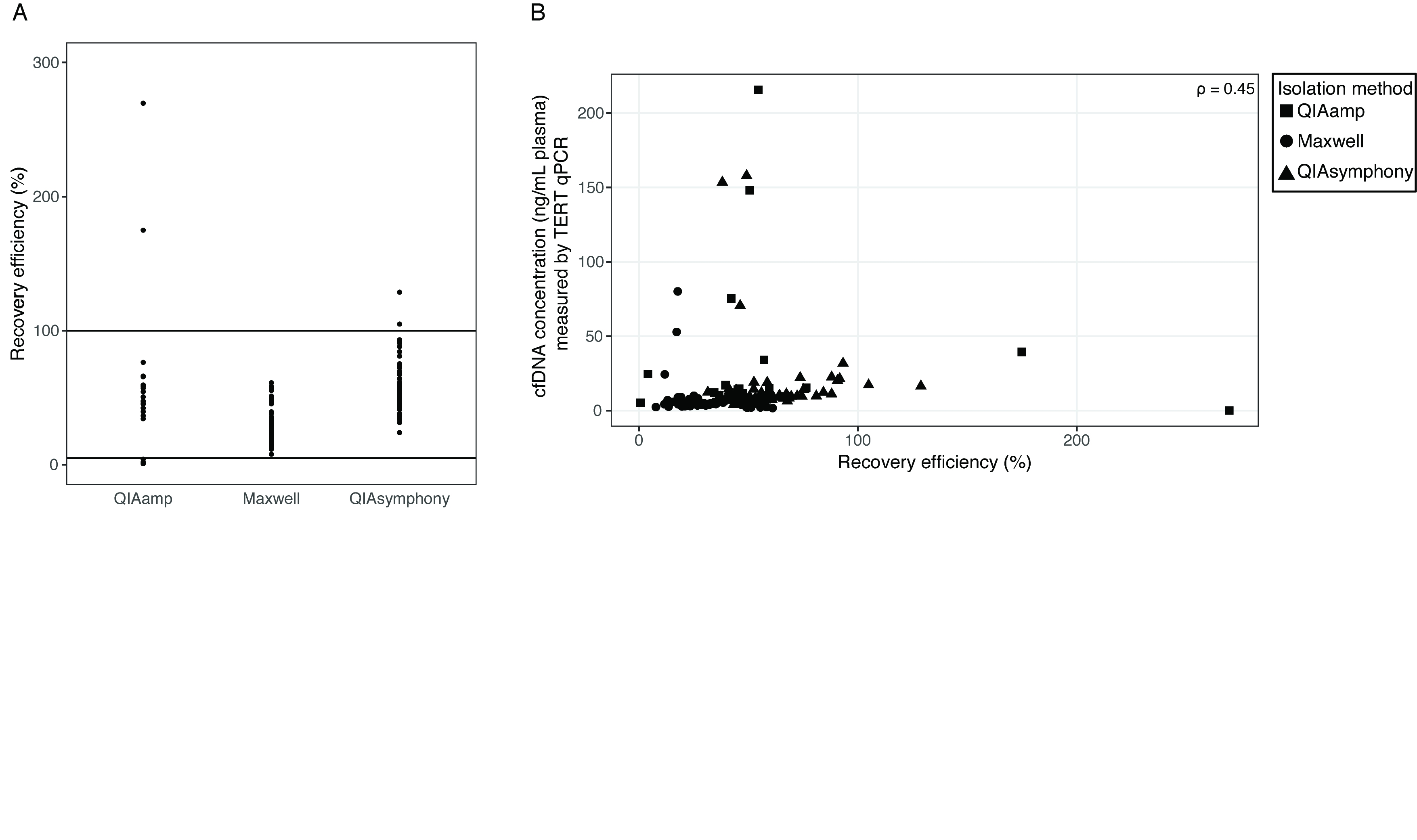

Supplement: Supplementary file 1 — Fig. S1. Overview of the recovery efficiency of synthetic plant DNA in all samples isolated with the different platforms (QA, MX, and QS). (A) Dot plot of the recovery efficiency for each isolation platform, as analyzed by plant qPCR using spiked‐in synthetic plant DNA. Samples with a recovery efficiency < 5% or > 100% (black horizontal lines) were excluded from the analyses. (B) Correlation between recovery efficiency and cfDNA concentration (ng·mL−1 plasma) measured by TERT qPCR assay. Correlations were tested by Spearman's rank correlation coefficient. *P < 0.001. [file MOL2-13-392-s001.tif]

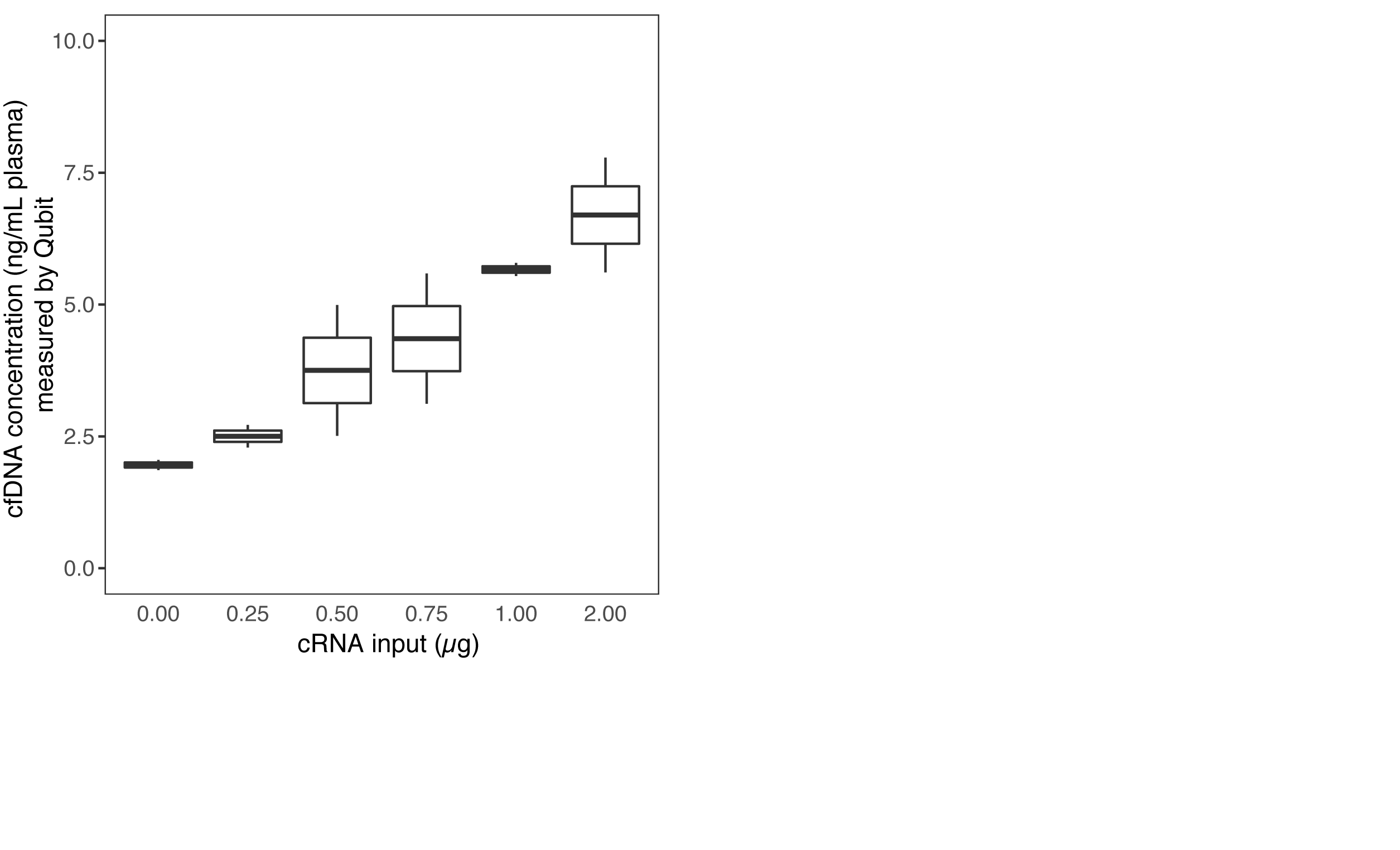

Supplement: Supplementary file 2 — Fig. S2. Effect of cRNA addition on cfDNA quantity using the QS platform. cfDNA concentration (ng·mL−1 plasma) was determined by Qubit after adding increasing amounts of cRNA (0–4 μg) before start of the plasma isolation. Boxes (interquartile ranges; IQR) and whiskers (1.5× IQR) are shown together with the median (black horizontal line). [file MOL2-13-392-s002.tif]

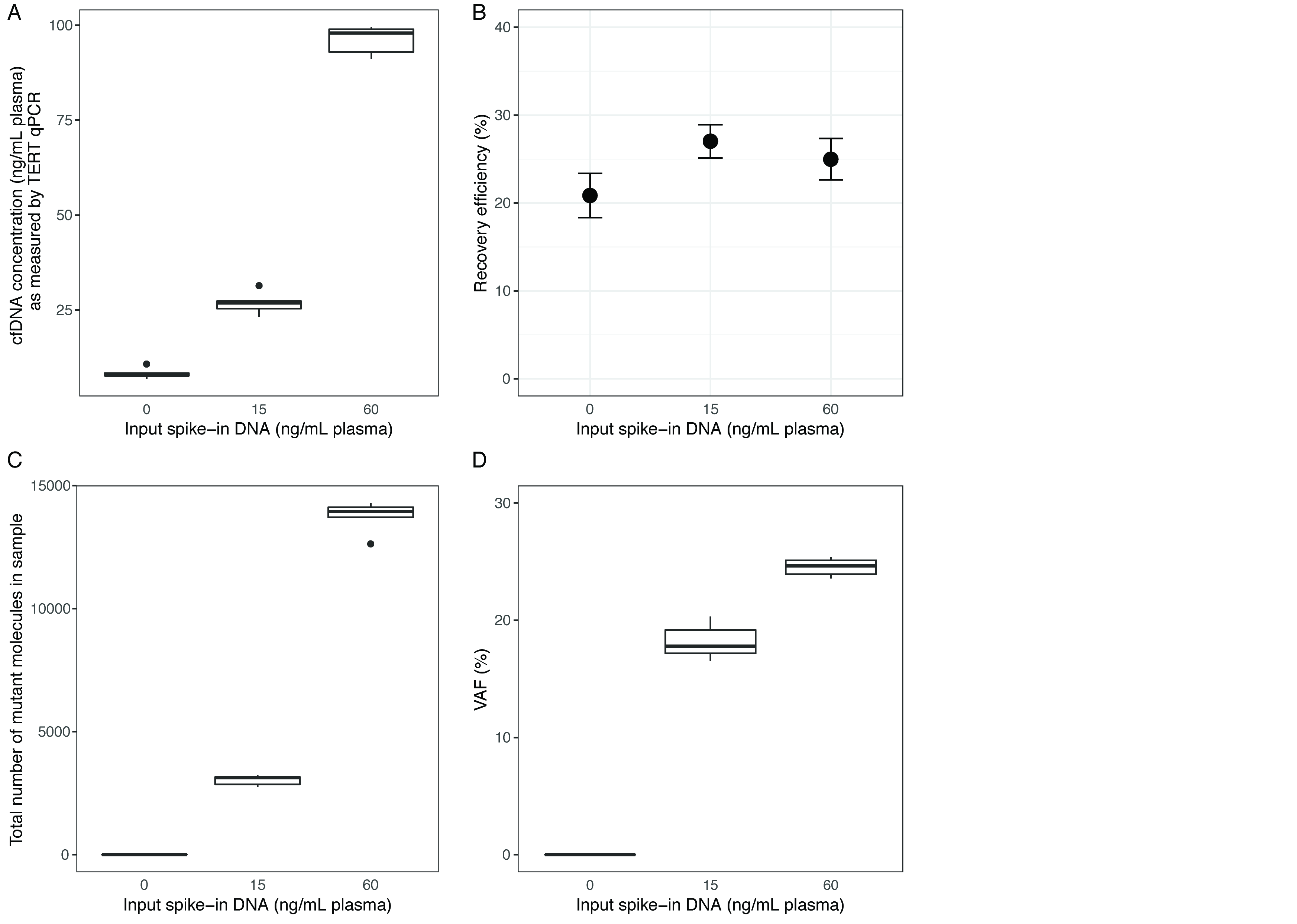

Supplement: Supplementary file 3 — Fig. S3. Performance of the MX platform using increasing DNA input (0, 15, and 60 ng·mL−1 fragmented cell line DNA has been spiked in HBD plasma). The effects on (A) cfDNA concentration (ng·mL−1 plasma) measured by TERT qPCR, (B) recovery efficiency measured by plant DNA qPCR, (C) total number of mutant molecules, and (D) VAF are shown. Boxes (interquartile ranges; IQR) and whiskers (1.5× IQR) are shown together with the median (black horizontal line). Outliers are indicated as single black points. Symbol ● is mean value shown with whiskers (standard deviation). N = 5. [file MOL2-13-392-s003.tif]

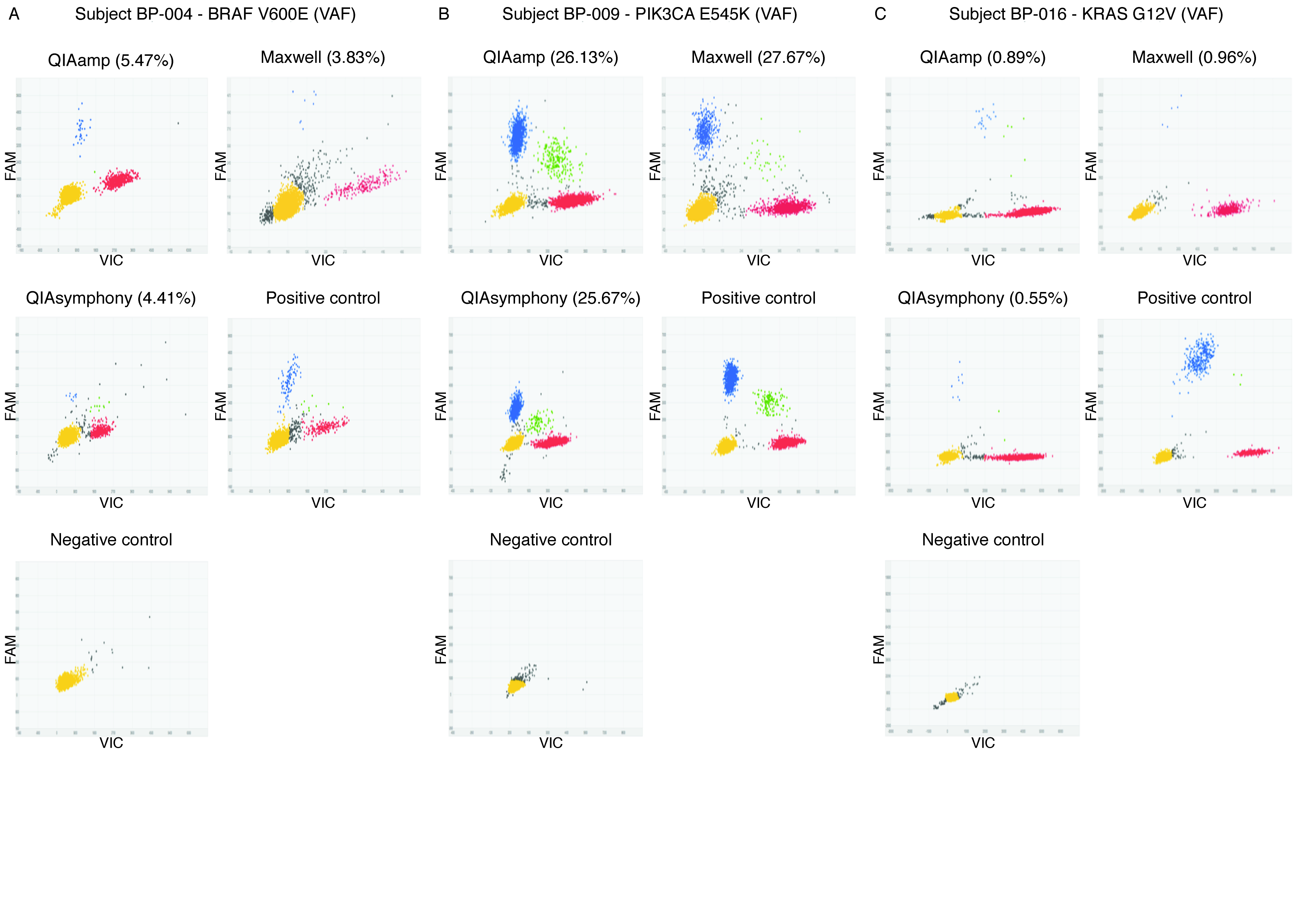

Supplement: Supplementary file 4 — Fig. S4. Representative data images of SNP genotyping dPCR assay isolated with the different platforms (QA, MX, and QS). A subject with an intermediate (A), high (B) and low (C) VAF are shown. On the Y‐axis, positive FAM signal represents mutant molecules (blue dots); on the X‐axis, positive VIC signal represents wild‐type molecules (red dots). Green dots reflect the presence of a mutant and a wild‐type molecule in a single well. [file MOL2-13-392-s004.tif]
