# Supplementary material for: High‐throughput isolation of circulating tumor DNA: a comparison of automated platforms
Source: Mol Oncol. 2018 Dec 22;13(2):392–402. doi: 10.1002/1878-0261.12415 (PMC6360376; doi:10.1002/1878-0261.12415)
Supplement: Supplementary file 5 — Table S1. Custom primer and probe sequences used for qPCR. Table S2. Standard SNP genotyping assays. Table S3. Custom SNP genotyping assays. [file MOL2-13-392-s005.docx]

**Supplemental tables**

| **Supplemental table 1. Custom primer and probe sequences used for qPCR.** | | | | | |
| --- | --- | --- | --- | --- | --- |
| **Assay Name** | **Forward primer** | **Reverse primer** | **Probe** | **Amplicon size (bp)** | **Manufacturer** |
| β-actin 136 bp | 5’-GCG CCG TTC CGA AAG TT-3’ | 5’- CGG CGG ATC GGC AAA -3’ | FAM-ACC GCC GAG ACC GCG TC-MGBNFQ | 136 | Invitrogen |
| β-actin 420 bp | 5’-CCG CTA CCT CTT CTG GTG-3’ | 5’-GAT GCA CCA TGT CAC ACT G-3’ | VIC-CCT CCC TCC TTC CTG GCC TC-BHQ | 420 | Invitrogen |
| β-actin 2000 bp | *The β-actin 2000 bp fragment is detected when both primers and probes of the β-actin 136 bp assay and the β-actin 420 bp are able to amplify a long DNA fragment. This double positive signal is detected by the QuantStudio 3D Digital PCR System (van Dessel et al., 2017).* | | | | Invitrogen |
| Plant | 5’-GAT CTT CAA CCA GGA GAT CA-3’ | 5’-AGT GAC AGT GAG GAC AAT CC-3’ | FAM-ACC CAT CTT CAC CGG A‐BHQ1 | 70 | Primers: IDT  Probe: Sigma-Aldrich, Saint Louis, Missouri, USA |

| **Supplemental table 2. Standard SNP genotyping assays.** | | | | | |
| --- | --- | --- | --- | --- | --- |
| **Assay ID** | **Assay Name** | **Gene** | **Cosmic ID** | **Amino acid change** | **Nucleotide change** |
| AHS1P6Q | NRAS_584 | *NRAS* | 584 | p.Q61R | c.182A>G |
| AHD2BW0 | KRAS_532 | *KRAS* | 532 | p.G13D | c.38G>A |
| AHABHHX | PIK3CA_763 | *PIK3CA* | 763 | p.E545K | c.1633G>A |

| **Supplemental table 3. Custom SNP genotyping assays.** | | | | | | | | | | |
| --- | --- | --- | --- | --- | --- | --- | --- | --- | --- | --- |
| **Assay ID** | **Assay Name** | **Gene** | **Cosmic ID** | **Amino acid change** | **Nucleotide change** | **Forward primer** | **Reverse primer** | **Probe** | **Amplicon size (bp)** | **Manufacturer** |
| ANNKR4W | BRAF_V600K_72bp | *BRAF* | 473 | V600K | c.1798_1799GT>AA | 5’-TCA TGA AGA CCT CAC AGT AAA AAT AGG T-3’ | 5’- TGG GAC CCA CTC CAT CGA-3’ | Variant: FAM-TGG TCT AGC TAC AAA GA-NFQ  Wild type: VIC- TTT TGG TCT AGC TAC AGT GA-NFQ | 72 | ThermoFisher Scientific |
| AN47WF2 | BRAF_V600E_72bp-2 | *BRAF* | 475 | V600E | c.1799_1800 TG>AA | 5’-TCA TGA AGA CCT CAC AGT AAA AAT AGG T-3’ | 5’-TGG GAC CCA CTC CAT CGA-3’ | Variant: FAM-TTG GTC TAG CTA CAG AAA-NFQ  Wild type: VIC- TTT TGG TCT AGC TAC AGT GA-NFQ | 72 |  |
| AN9HJKW | KRAS_G12C_76bp | *KRAS* | 516 | G12C | c.34G>T | 5′-TGC TGA AAA TGA CTG AAT ATA AAC TTG TG-3’ | 5′- AGC TGT ATC GTC AAG GCA CTC TT-3’ | Variant: FAM-TTG GAG CTT GTG GCG TA-NFQ  Wild type: VIC- TTG GAG CTG GTG GCG T-NFQ | 76 |  |
| ANU63FK | KRAS_G12D_76bp | *KRAS* | 521 | G12D | c.35G>A | 5′-TGC TGA AAA TGA CTG AAT ATA AAC TTG TG-3’ | 5′-AGC TGT ATC GTC AAG GCA CTC TT-3’ | Variant: FAM-TTG GAG CTG TTG GCG TA-NFQ  Wild type: VIC-TTG GAG CTG GTG GCG T-NFQ | 76 |  |
| ANAAAYM | KRAS_G12V_76bp | *KRAS* | 520 | G12V | c.35G>T | 5′-TGC TGA AAA TGA CTG AAT ATA AAC TTG TG-3’ | 5′-AGC TGT ATC GTC AAG GCA CTC TT-3’ | Variant: FAM-TTG GAG CTG ATG GCG TA-NFQ  Wild type: VIC-TTG GAG CTG GTG GCG T-NFQ | 76 |  |
